# Comparing rolling resistance of two treadmills and its influence on exercise testing in wheelchair athletics

**DOI:** 10.3389/fpsyg.2022.1085553

**Published:** 2023-01-06

**Authors:** Ursina Arnet, Fabian Ammann, Claudio Perret

**Affiliations:** ^1^Swiss Paraplegic Research, Nottwil, Switzerland; ^2^Institute of Sports Medicine, Swiss Paraplegic Center, Nottwil, Switzerland

**Keywords:** testing, equipment, reproducibility, athletes, wheelchair

## Abstract

Standardized laboratory exercise testing is common in sport settings and rehabilitation. The advantages of laboratory-based compared to field testing include the use of calibrated equipment and the possibility of keeping environmental conditions within narrow limits, making test results highly comparable and reproducible. However, when using different equipment (e.g., treadmills), the results might deviate and impair comparability. The aim of this study was to compare the biomechanical properties (rolling resistance, speed, inclination) of two treadmills regularly used for exercise testing in elite wheelchair athletes. During the experiment, speed and inclination of two treadmills (same model and producer, different manufacturing year and belt material) were verified. Standardized drag tests were performed to assess rolling resistance. Power output conducted by the athlete during later exercise tests was calculated based on the results. Speed and inclination deviated only slightly from the values indicated by the producer. Rolling resistance caused by different belt material was mainly accountable for the differences in power output between the treadmills. In general, athletes had to deliver 10% more power output on one of the treadmills compared to the other. Concluding from these results: if different treadmills are used for testing, a proper validation is recommended to avoid misleading interpretations of test results.

## 1. Introduction

Standardized laboratory exercise testing is common in rehabilitation and sport settings whereas numerous testing methods and protocols are applied. Common tests with wheelchair athletes are the lactate minimum test (Perret et al., 2012) or the VO_2_ max test (Leicht et al., 2013). These tests indicate the endurance exercise capacity and are a helpful tool to determine training intensity zones and to guide the training process. The advantages of laboratory-based compared to field testing include the use of calibrated equipment and the possibility of keeping environmental conditions (e.g., temperature, humidity) within narrow limits, making test results highly comparable and reproducible. Especially in elite sports, such characteristics are of utmost interest to detect minimal performance differences, as small time differences of less than 0.5% of the racing time decide over winning or losing a medal at international championships, such as Paralympic Games (Perret, 2017). Typical diurnal fluctuations of performance are commonly at around 1% of the time trial performance (Fiedler et al., 2022).

In wheelchair athletics standardized endurance exercise testing is often performed on a treadmill (Perret et al., 2012). Ideally, these tests are always performed on the same treadmill and under the same environmental conditions to make test results as comparable as possible. However, this prerequisite seems not always to be given as athletes from a national team often train and test at different locations. In order to warrant a high measurement quality as well as a fair comparison of test results between athletes, regular quality controls of the equipment seems therefore highly recommended. In fact, some years ago a study compared several treadmills which were used for exercise testing in with a spinal cord injury in eight Dutch rehabilitation centers (de Groot et al., 2006). Although the exactly same type of treadmill was used in seven of eight centers, the standardized wheelchair drag tests revealed significant differences between different locations. Treadmill speed, inclination and rolling resistance seemed to be the most critical factors which have to be taken into account.

Recently, our institution replaced the treadmill for exercise testing of elite wheelchair racing athletes. This device was bought to replace the former, exactly same type of treadmill from the same company. However, being aware of the pitfalls found in the above-mentioned study in a rehabilitation setting (de Groot et al., 2006), a critical investigation comparing the two devices seemed to be reasonable to avoid uncertainty of our athletes based on potential misinterpretations of test results. Thus, the aim of the present study was to compare the biomechanical properties (rolling resistance, speed, inclination) of two treadmills regularly used for exercise testing in elite wheelchair athletes under standardized controlled conditions. We hypothesize that rolling resistance, speed and inclination of the two treadmills are consistent.

## 2. Materials and methods

Two treadmills were compared. Treadmill A: Cosmos Saturn, HP Cosmos, Traunstein, Germany with a black belt (width 1 m, length 2.5 m), year of manufacture 2005. Treadmill A was in use for 16 years. Treadmill B: Cosmos Saturn, HP Cosmos, Traunstein, Germany with a green belt (width 1.25 m, length 3 m), year of manufacture 2020. Treadmill B was in use for 1 year. Data has been analyzed descriptively to address the research question.

### 2.1. Speed

**Figure 1:**
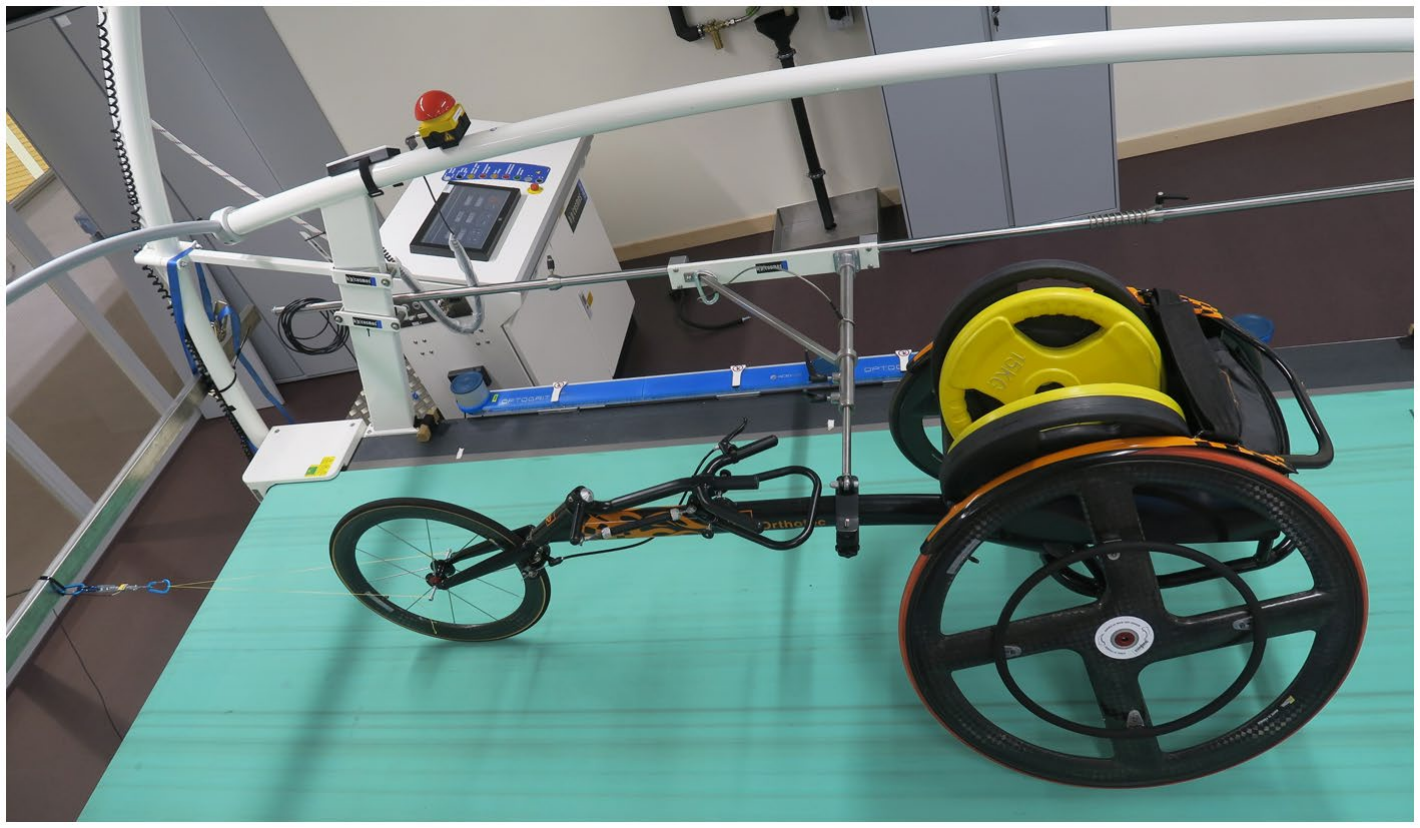
Wheelchair on the treadmill, loaded with a weight of 80 kg for the drag test, attached to the fixation system alongside of the treadmill and to the force sensor in front.

The speed of the two treadmills was compared with and without a racing wheelchair (Eliminator OSR Racing, Top End, tire pressure of 8 bar, loaded with a weight of 80 kg) driving on the treadmill, while the treadmill had an inclination of 0 and 10%. The wheelchair was attached to a fixation system, which slides alongside the treadmill and holds the wheelchair in a secured position (Figure 1). The time duration of 50 complete revolutions of the treadmill belt was measured. The following speeds were revised: 10, 15, 20 and 25 km/h.

### 2.2. Inclination

The inclination of the treadmill belt was measured at 0 to 10% (steps of 1%) with a digital inclinometer (PRO 360, SPI, Garden Grove, United States) during all drag tests to assess rolling resistance.

### 2.3. Rolling resistance

A systematic set of drag tests was performed to assess rolling resistance (van der Woude et al., 1986). During the drag tests, the racing wheelchair was attached with a rope to a force sensor (Futek model LSB200, Futek, Irvine, United States). To keep the wheelchair on track, it was secured with a fixation system (Figure 1). The drag test was performed for both treadmills with six different loading conditions (Table 1). The loading conditions were chosen to represent a spectrum of possible testing conditions, e.g., different weight of the athletes (condition 1 and 2 vs. condition 3–6), different tire pressure (condition 1, 3 and 5 vs. condition 2, 4 and 6). In addition to the standardized weights placed in the wheelchair (condition 1–4) we performed the drag test with an athlete (condition 5 and 6), whose weight was corresponding to condition 3 and 4. The participant was a wheelchair athlete with a spinal cord injury (33 years, 80.5 kg). The wheelchair used for the experiment was the personal racing wheelchair of the participant and thus well fitted to the participants anthropometry.

**Table 1:** Different conditions at which the drag test was performed.

Condition	Load	Weight [kg]	Tire pressure [bar]
1	Weights	50.9	6
2	Weights	50.9	8
3	Weights	81.9	6
4	Weights	81.9	8
5	Participant	80.5	6
6	Participant	80.5	8

Each drag test was performed at a speed of 4 km/h and at inclinations from 0 to 10% (steps of 1%) according to van der Woude et al. (1986). At level treadmill, the measurement of the drag force might be unstable. Therefore, drag force at 0% inclination was not measured directly, but determined through extrapolation *via* a linear regression analysis (van der Woude et al., 1986).

### 2.4. Resulting power output for athlete

With the calculated drag force, the measured speed and inclination of the treadmills we calculated the power output which has to be conducted by the athlete during later exercise tests to meet the test conditions. Typical exercise conditions of 2% inclination and speeds of 10 km/h, 20 km/h and 30 km/h were chosen.

## 3. Results

### 3.1. Speed

The speed of the treadmill was not affected by the different conditions (with/without wheelchair on treadmill at 0 and 10% inclination). The mean of the speed measured at the different conditions of both treadmills is indicated in Table 2. The difference between the measured speed and the speed indicated at the treadmill was between 0.3% and 1.1% of the indicated speed.

**Table 2:** Mean of measured speed of both treadmills (A, B).

Speed indicated [km/h]		10	15	20	25
A	Measured [km/h]	10.08	15.17	20.20	25.26
B	Measured [km/h]	10.04	15.05	20.06	25.07

### 3.2. Inclination

The inclinations of both treadmills are listed in Table 3. Differences between treadmills were maximally 0.1°. The difference between the measured inclination and the inclination shown at the treadmill was between −0.2° and 0.1°. Predefined angles were set to 0.57° (1% of inclination) per step. The step size of the actual slope varied between 0.5° and 0.6°, which is within the accuracy of the inclinometer. Only one step size of treadmill A was 0.7° and thus slightly deviating from the intended step size of 0.57°.

**Table 3:** Measured inclination in degree of both treadmills (A, B) for the indicated inclination.

Incli-nation	0° (0%)	0.6° (1%)	1.1° (2%)	1.7° (3%)	2.3° (4%)	2.7° (5%)	3.4° (6%)	4.0° (7%)	4.6° (8%)	5.1° (9%)	5.7° (10%)
A	−0.2°	0.5°	1.0°	1.6°	2.2°	2.7°	3.3°	3.9°	4.4°	5.0°	5.5°
B	−0.1°	0.5°	1.1°	1.7°	2.2°	2.8°	3.4°	3.9°	4.5°	5.1°	5.6°

### 3.3. Rolling resistance

**Figure 2:**
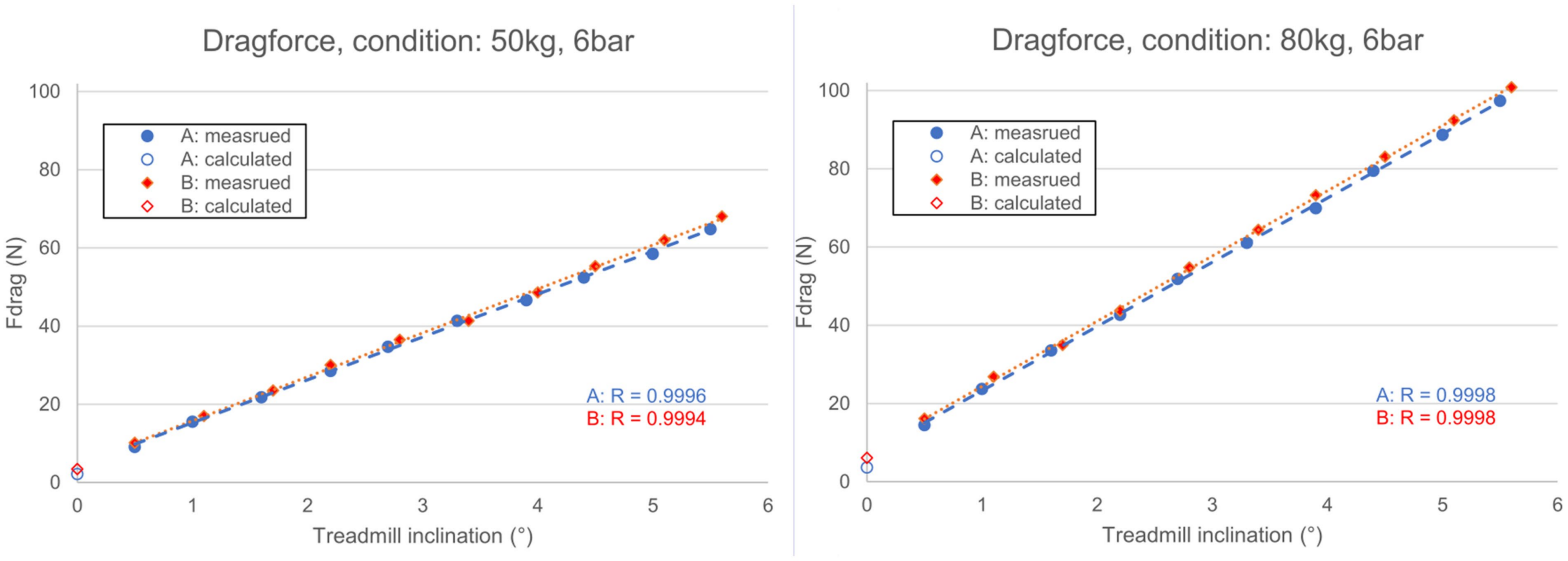
Measured and calculated drag forces, obtained from the drag test.

The correlation coefficient of the linear regression to determine drag force at 0° was very high (0.9996–0.9999). Measured and calculated drag forces of condition 1 and 3 are displayed in Figure 2. All results of the drag tests are listed in Table 4.

**Table 4:** Results of the drag test comparing both treadmills (A, B): calculated rolling resistance (Fdrag) at the level treadmill, and calculated power output at the conditions used during later exercise testing with athletes (inclination of 2%, speed of 10 km/h, 15 km/h, 20 km/h and 25 km/h).

Condition	Treadmill	Fdrag	Power output at 2% incline			
			10 km/h	15 km/h	20 km/h	25 km/h
1: 50 kg, 6 bar	A	4.3 N	42.8 W	64.4 W	85.8 W	107.3 W
	B	4.6 N	47.2 W	70.7 W	94.3 W	117.8 W
2: 50 kg, 8 bar	A	4.9 N	41.5 W	62.4 W	83.1 W	104.0 W
	B	4.4 N	46.7 W	70.1 W	93.4 W	116.7 W
3: 80 kg, 6 bar	A	6.9 N	65.2 W	98.1 W	130.6 W	163.4 W
	B	7.7 N	72.6 W	108.8 W	145.1 W	181.3 W
4: 80 kg, 8 bar	A	7.2 N	66.2 W	99.6 W	132.6 W	165.8 W
	B	7.4 N	71.9 W	107.7 W	143.6 W	179.4 W
5: participant, 6 bar	A	5.8 N	60.9 W	91.7 W	122.1 W	152.7 W
	B	6.8 N	69.2 W	103.8 W	138.3 W	172.8 W
6: participant, 8 bar	A	4.3 N	61.8 W	93.0 W	123.8 W	154.8 W
	B	6.2 N	68.3 W	102.4 W	136.5 W	170.6 W

## 4. Discussion

The comparison of the two treadmills (same brand, same mode, but different belt material) showed that slight differences exist between the treadmills, and that mainly belt properties result in noticeable differences for the athlete when doing a performance test.

For both treadmills, speed and inclination only deviate marginally from the indicated speed and inclination. Differences between indicated and measured speed and inclination reported by de Groot et al. were higher than measured in this study (de Groot et al., 2006). When measuring actual belt velocity of 7 identical Bonte treadmills running at 2 km/h, de Groot et al. reported values of 1.5 km/h to 1.9 km/h. This is a maximal difference of 20% of intended velocity. In our comparison of two Cosmos Saturn Treadmills, the maximal difference was 0.8% of the intended velocity. Regarding inclination, de Groot et al. measured step sizes of 0.22° to 0.42° when aiming at 0.36° per step. This is a higher difference than measured in our study, where we found step sizes of 0.5° to 0.6° when aiming at 0.57° per step. Thus, compared to previous studies, the deviation of speed and inclination of the two treadmills compared in our study is small.

Rolling resistance varies between the two treadmills, likely resulting from different belt material. From previous studies it is known that the surface accounts for a high variance in rolling resistance (Ott and Pearlman, 2021). For example, carpet has approximately 3 times higher rolling resistance than concrete or linoleum (Hoffman et al., 2003; Sauret et al., 2012). The different belt material might also account for the different reaction on change in tire pressure. Increasing tire pressure in the standardized conditions (wheelchair loaded with given weight) from 6 to 8 bar results in an increased rolling resistance on treadmill A and in a decreased rolling resistance on treadmill B. A decrease in rolling resistance has been seen earlier when increasing tire pressure on a manual wheelchair (Pavlidou et al., 2015). When the participant was sitting in the wheelchair, the reaction on increasing tire pressure was reversed. This change might be related to differences in weight distribution and the resulting change in the location of center of mass. The weights were placed into the seat of the racing wheelchair; therefore, center of mass was located more toward the back of the wheelchair and the back wheels placed more pressure onto the belt. When the participant was sitting in the wheelchair, he placed his hand on the steering mechanism of the wheelchair. This will result in a forward shift of the center of mass and even a small change in mass distribution can have a significant impact on rolling resistance (Ott and Pearlman, 2021).

Considering all the differences and slight deviations it results in a noticeable difference for the athlete when doing a performance test. At a lower speed of 10 km/h, lighter athletes (50 kg) have to deliver about 5 W more on treadmill B compared to treadmill A in order to keep up with the speed. Heavier athletes (80 kg) have to deliver 7 W more on treadmill B compared to A at the same condition. At faster conditions (25 km/h) the differences are even higher. Lighter athletes have to deliver approximately 11 W more on treadmill B compared to A, for heavier athletes it results in a mean difference of 16 W. In general, athletes have to deliver 10% more power output on treadmill B compared to treadmill A (Table 4).

### 4.1. Practical implications

Today, medal decisions at international competitions such as Paralympic Games or World Championships lie within a split second (Perret, 2017). Therefore, reliable exercise testing procedures and results have to be warranted for athletes, coaches and exercise physiologists to make clear statements and give correct and feasible training advices. The present investigation showed that two treadmills from the same manufacturer used under comparable conditions (e.g., same speed, incline, weight and tire pressure) even resulted in considerable differences. A limitation of the present study is that comparisons made in the present study are based on one specific treadmill model. However, results from previous studies have shown similar or higher differences for other treadmill models (de Groot et al., 2006). The differences found in both studies are much higher than an expected error of measurement or the daily performance fluctuation (Fiedler et al., 2022). Thus, beside the regular quality management routine of an exercise testing laboratory and stable environmental conditions (temperature, humidity) it is highly recommended to keep also an eye on the athletes’ equipment and to use always the same tire pressure. In addition, the exactly same testing device with a standardized setting has to be used. If a new treadmill is installed, a proper validation is recommended before athletes are tested to avoid misleading interpretations of test results. Finally, athletes and coaches have to be sensitized that the use of different devices at different locations my lead to different results and has to be avoided.

### 4.2. Conclusion

The discrepancies between the two Cosmos Saturn treadmills resulted in different calculated power outputs at given conditions. Speed and inclination deviated only slightly from the values indicated by the manufacturer and therefore did not contribute much to the change in power output. It was mainly the rolling resistance caused by the different belt material that was accountable for the differences in power output between the treadmills. In order to draw meaningful conclusions from performance tests, athletes should always be measured on the same treadmill using the same tire pressure. If different treadmills are used for testing, a proper validation is recommended in advance to avoid misleading interpretations of test results.

## Data availability statement

The original contributions presented in the study are included in the article/supplementary material, further inquiries can be directed to the corresponding author.

## Ethics statement

Ethical review and approval was not required for the study on human participants in accordance with the local legislation and institutional requirements. The patients/participants provided their written informed consent to participate in this study.

## Author contributions

UA, CP, and FA initiated the study and contributed to the conception and design of the study. UA and FA performed the data collection. UA was responsible for all analyzes, drafting, and finalization of the paper. All authors critically revised the paper and have read and approved the final paper.

## Funding

This study was funded by Institute of Sports Medicine, Swiss Paraplegic Center: open access publication fee.

## Conflict of interest

The authors declare that the research was conducted in the absence of any commercial or financial relationships that could be construed as a potential conflict of interest.

## Publisher’s note

All claims expressed in this article are solely those of the authors and do not necessarily represent those of their affiliated organizations, or those of the publisher, the editors and the reviewers. Any product that may be evaluated in this article, or claim that may be made by its manufacturer, is not guaranteed or endorsed by the publisher.
